# Cognitive Changes in Laparoscopic Cholecystectomy: Cognitive Assessments in Decision-Making Matters

**DOI:** 10.3390/jcm15124569

**Published:** 2026-06-12

**Authors:** Carolina Mello, Sergio Schmidt

**Affiliations:** 1 Department of Surgery, Anesthesiologist, Federal University of the State of Rio de Janeiro, Rio de Janeiro 21941-901, Brazil; carolmello77@gmail.com; 2 Post-Graduate Program in Neurology, PPGNEURO, Federal University of the State of Rio de Janeiro, Rio de Janeiro 21941-901, Brazil

**Keywords:** anesthesia, attention, neuroinflammation, postoperative cognitive dysfunction, propofol, sevoflurane, anesthesia, intravenous, anesthesia, inhalation

## Abstract

**Background:** Cognitive changes after minor surgery may affect patient safety, functional recovery, and readiness for hospital discharge, even after low-risk procedures with early discharge protocols. In this regard, patients require neuropsychological assessment at discharge, which may have important clinical implications for return to daily activities and postoperative decision-making. Our study investigated postoperative cognitive changes after minor surgery under general anesthesia using a neuropsychological assessment and a non-surgical group. **Methods:** Patients undergoing laparoscopic cholecystectomy received propofol or sevoflurane anesthesia. A non-surgical control group was included. Cognitive performance was assessed at baseline and discharge using the Computerized Visual Attention Test (CVAT), the controlled oral word association test (COWAT), and the symbol digit modalities test (SDMT). Relative change scores were calculated as ((baseline − postoperative performance)/baseline). Group differences were analyzed using two-tailed multivariate analysis of variance (MANOVA), followed by ANOVAs and Bonferroni-adjusted pairwise comparisons. **Results:** A total of 105 participants were included (37 non-surgical, 34 propofol, 34 sevoflurane). MANOVA showed a significant group effect (Pillai’s trace = 0.332, *p* < 0.001, η^2^ = 0.196). The ANOVAs revealed significant differences in sustained attention (CVAT), verbal fluency (COWAT) and executive function (SDMT). The propofol group showed evident decline in sustained attention compared to non-surgical. In verbal fluency, non-surgical improved at day after, whereas both surgical groups showed no improvement, indicating worse performance. In SDMT the sevoflurane group had worse performance. **Conclusions:** Minor surgery under general anesthesia may lead to transient impairments in attention and learning at discharge, supporting the need for postoperative cognitive monitoring and individualized discharge decisions.

## 1. Introduction

Although cognitive deficits have been observed during major surgeries since 1887, the underlying mechanisms remain incompletely understood [1]. The occurrence of specific cognitive deficits after minor surgical operations has been reported and can potentially interfere with patient safety and functional recovery during the immediate postoperative period [1,2,3,4]. Those alterations may affect attention subdomains, executive functioning, psychomotor performance, and learning capacity, domains directly involved in activities required after hospital discharge [1,2,3,4]. The importance of this research is emphasized by the exponential increase in elective surgical procedures occurring every decade, projected to reach 5000 operations per 100,000 patients by 2030 [5]. In this context, laparoscopic cholecystectomy, due to its established safety profile and cost-effectiveness, is performed as ambulatory surgery in 90% of cases, typically with same-day discharge (SDD) [6]. All these discussions’ trends highlight the need for cognitive assessment at hospital discharge [6].

Cognitive impairments can still happen due to the multifactorial pathophysiology [2]. Neuroinflammation and disruption of the blood–brain barrier have been proposed as central features [2,7]. It raises questions regarding their underlying mechanisms [2,3,4]. Anesthesiologists may reduce these risks with clinical personalized intraoperative anesthesia decisions, by maintaining physiological homeostasis and preventing intraoperative hypotension, hypoxemia, hypothermia, and excessive anesthetic depth [2,8]. Nevertheless, cognitive impairments can still happen due to the multifactorial pathophysiology [2]. Neuroinflammation and disruption of the blood–brain barrier have been proposed as central features [2,8].

Different anesthetic techniques have been investigated as potential modulators of postoperative cognitive outcomes. Propofol, an intravenous drug, has been shown to induce anti-inflammatory and antioxidant functions that may have neuroprotective effects in humans [9,10]; sevoflurane, an inhalant, has been linked with cognitive impairments, possibly because of its pro-inflammatory and neurotoxic activities [9,10]. These hypotheses have inspired several prospective randomized controlled trials and systematic reviews and meta-analyses comparing propofol and sevoflurane anesthesia. Although other studies indicate greater positive effects of propofol on cognitive impairments [11], their findings are inconclusive, owing to methodological limitations such as heterogeneity in cognitive testing and anesthesia protocols [12,13,14].

In this rationale, the present study was conducted to investigate postoperative cognitive changes after minor laparoscopic surgery under general anesthesia, using a non-surgical group to control for the learning effects associated with repeated neuropsychological testing. We compared preoperative baseline cognitive performance with performances on the discharge day as the primary outcome, and at 15 and 45 days as secondary outcomes. It was hypothesized that minor surgical procedures might be associated with cognitive changes and that propofol would protect against cognitive changes at hospital discharge.

**Figure 1:**
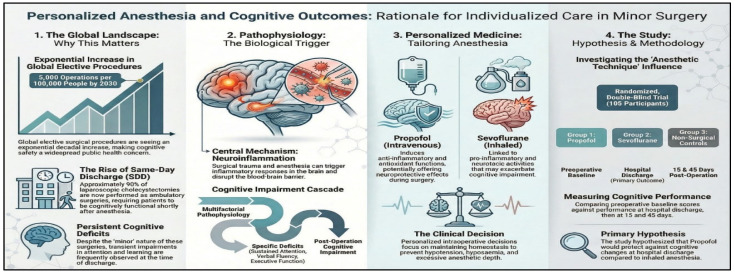
A schematic representation of rationale of this study. Created using NotebookLM^®^ (Google, CA, USA, accessed on 9 March 2026).

The rationale of this study is explained in Figure 1.

## 2. Materials and Methods

### 2.1. Ethics

The study was prospectively registered with the Brazilian Clinical Trials Registry (ReBEC) (RBR-6xhp399; UTN code: U1111-1295-3640, approved on 8 August 2023) and was conducted at the Gaffrée and Guinle University Hospital of the Federal University of the State of Rio de Janeiro. The trial protocol was approved by the Research Ethics Committee of the same institution (CAAE: 54581221.0.0000.5258; Approval No.: 5.187.287 approved on 27 December 2021). The data were collected from September 2023 to September 2024. The present report is a partial analysis of a larger prospective research project.

### 2.2. Study Design

We conducted a single-center, randomized, controlled, double-blind, two-arm, parallel-group clinical trial in accordance with the Ethical Principles for Medical Research Involving Human Subjects outlined in the Declaration of Helsinki. The institutional review board approved the study, and written informed consent was obtained from all participants. This manuscript adheres to the Consolidated Standards of Reporting Trials (CONSORT) 2025 guidelines for randomized controlled trials, and we used generative artificial intelligence (GenAI) to generate a graphic abstract and the figures included in this study; specifically, NotebookLM^®^ (Google, based on large language models, accessed on 9 March 2026) was employed to create conceptual figures based on the authors’ input and scientific content. All Gen AI images were critically reviewed, edited and validated by the authors to ensure accuracy and consistency with the scientific data presented.

Two well-established general anesthesia techniques, a propofol-based, total intravenous anesthesia (propofol group) and a sevoflurane-based, inhalation anesthesia (sevoflurane group), were compared to evaluate postoperative cognitive impairment. Surgical patients were randomly assigned to one of these two interventions. A non-randomized, non-surgical comparative group was included in the cohort to assess baseline cognitive performance as a control to allow differentiation between true postoperative changes and learning effects associated with repeated neuropsychological testing. This non-surgical group consisted of healthy volunteers (companions or relatives of the surgical patients) who met the same eligibility criteria as the surgical groups but were not exposed to anesthesia, surgery, perioperative pain, or hospitalization-related physiological stress factors. Although not randomized, the inclusion of this cohort provided an important methodological reference for interpreting postoperative cognitive trajectories over time and strengthened the identification of cognitive changes specifically associated with the perioperative period.

### 2.3. Participants

Eligible participants were adults older than 18 years with an ASA physical status of I or II who were scheduled for elective laparoscopic cholecystectomy under general anesthesia. Non-inclusion criteria included: ASA physical status III or IV; neurological or psychiatric disorders affecting cognition; chronic use of corticosteroids, antipsychotics, opioids, zolpidem, or tricyclic antidepressants; smoking or alcoholism; severe hepatic disease; renal insufficiency; inability to complete neuropsychological testing; postoperative delirium; perioperative protocol complications such as hemorrhage, hypoxemia, or hypotension; excessive anesthetic depth (defined as a significant number of intraoperative Bispectral Index [BIS] values ≤ 35); previous general anesthesia within the prior year; prior cardiac or neurological surgery; surgical duration exceeding 300 min; emergency surgery; or major intraoperative surgical complications.

### 2.4. Randomization, Group Allocation, and Blinding

Prior to participant enrolment, an independent researcher generated a computer-based randomization sequence using a 1:1 allocation ratio. Allocation concealment was ensured using sequentially numbered, opaque, sealed envelopes, which were opened by the anesthesiologist responsible for administering general anesthesia on the day of surgery.

Blinding was achieved by ensuring that the investigators who conducted the preoperative and postoperative neuropsychological assessments were different from those responsible for intraoperative anesthetic management. Both the patients and outcome assessors were blinded to group allocation. The anesthetic procedures were performed by four anesthesiologists working in collaboration with three separate surgical teams.

### 2.5. Cognitive Assessments

In our study, we used three tests: 1. the controlled oral word association test (COWAT) [15] 2. the symbol digit modalities test (SDMT) [16,17] and 3. the Computerized Visual Attention Test (CVAT) [18]. The assessments were conducted at four predefined time points: 1 day before surgery (baseline performance); on the day of hospital discharge (12–24 h postoperatively); 15 days after surgery (postoperative days 12–17) (D15); and 45 days after surgery (postoperative days 43–48) (D45). The same three neurocognitive tests were administered at each time point.

#### 2.5.1. COWAT

The COWAT in the phonemic mode [15] measured verbal fluency and executive function. In the phonemic (FAS task), participants were instructed to produce as many words as possible beginning with the letters F, A, and S within 60 s for each letter, except for proper nouns, numbers, and morphological variations. The variable is the total number of correct answers. The COWAT is a validated tool and its sensitivity to frontal lobe dysfunction has been proved [15].

#### 2.5.2. SDMT

Processing speed, fine motor coordination, and executive function were assessed using the SDMT [16,17]. Participants were instructed to match numbers to geometric symbols using a reference key within a 90 s time frame, with responses provided in written form. The variable is the total number of correct answers. To reduce learning effects and improve test–retest reliability, an alternative version of the SDMT was administered at baseline, on the day of discharge, and at D15, whereas the version administered at D45 was identical to that used at baseline [16,17].

#### 2.5.3. CVAT

**Figure 2:**
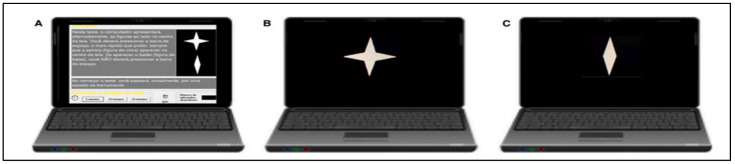
Provides a schematic overview of the CVAT task (target vs. non-target stimuli). Note: A schematic overview of the CVAT showing the target (star) and non-target (diamond). The test was conducted using a 13-inch laptop computer display (Windows^®^ 10; maximum timing error: 30 ms) in a quiet room with only the participant and the examiner present. The subjects were seated approximately 50 cm from the screen and were required to have 20/30 visual acuity, with corrective lenses permitted [19]. The CVAT begins with written instructions displayed on the screen (**A**): “In this test, the computer alternately displays the indicated figures in the center of the screen. You must press the spacebar using your dominant hand as fast as you can whenever the star appears in the center of the screen. If the other figure appears, you should not press the spacebar.” The target (**B**) remains on the screen for 250 ms. The non-target (**C**) also remains on the screen for 250 ms. The test consisted of 120 trials (one figure presented at a time, either target or non-target). The interstimulus interval varied between 1, 2, and 4 s and was equally distributed throughout the test. The total test duration was 5 min. Variables: OEs, CEs, RT, and VRT (standard deviation of the RTs during the test). The CVAT is available for research and clinical use by licensed psychologists upon request, from Prof. Sergio L. Schmidt. Versions are available in English, Spanish, and Portuguese. CVAT: Continuous Visual Attention Test [18].

The CVAT was used to assess attention subdomains (Figure 2) [18]. In this study, we administered the 5 min version of CVAT with 120 stimuli, including 60 target and 60 non-target geometric shapes. In response to target stimuli, participants were instructed to press the spacebar as quickly as possible [18]. CVAT is an evidence-based instrument designed to measure multiple aspects of attention. To evaluate cognitive performance, we used four variables: omission errors (OE), commission errors (CE), corrected reaction time (RT) and corrected variability reaction time (VRT). Poor performance may manifest as OE, defined as number of failures to respond to target stimuli and deficits in focus attention [19,20,21,22,23,24]; CE, defined as number of incorrect responses to non-target stimuli, representing impaired inhibitory control and increased impulsivity [19,20,21,22,23,24]; RT, defined as time of reaction to target stimuli response, in milliseconds (ms), indicating slower visuomotor processing speed and reduced alertness [19,20,21,22,23,24]; and intra-individual VRT, defined as standard deviation of RT, is the time of reaction to each target stimuli response in milliseconds, an increase in which is consistent with impaired sustained attention [19,20,21,22,23,24].

### 2.6. Anesthetic Management

General anesthesia was routinely administered in all surgical cases to avoid confounding related to inflammatory modulation. No anxiolytic or other preoperative medications were administered. All the patients received standard monitoring, including BIS monitoring. Anesthesia was induced with intravenous propofol (1.0–2.0 mg·kg^−1^) and a continuous infusion of remifentanil (0.10–0.5 µg·kg^−1^·min^−1^). Maintenance of anesthesia was achieved with continuous manual intravenous propofol (50–100 µg·kg^−1^·min^−1^) in the propofol group or inhaled sevoflurane (1–2% minimum alveolar concentration) in the sevoflurane group. Anesthetic doses were clinically personalized in both groups to target a BIS value between 45 and 55. Tracheal intubation was facilitated with rocuronium (0.6–1.2 mg·kg^−1^), and supplemental doses (10–20% of the initial dose) were administered every 30 min as needed until the end of surgery, guided by train-of-four monitoring, and sugammadex administered, when necessary. Tracheal extubation was performed in the operating room, and all the patients were subsequently transferred to the post-anesthesia care unit. Hemodynamic and ventilatory parameters were closely regulated in accordance with a predefined intraoperative protocol to maintain intraoperative homeostasis.

Postoperative analgesia followed a clinical personalized multimodal regimen, including intravenous metamizole (50 mg·kg^−1^), surgical wound infiltration, and a transversus abdominis plane (TAP) block with bupivacaine (1.0–2.0 mg·kg^−1^). Intravenous tramadol (50–100 mg) was administered as rescue analgesia when the visual analog scale (VAS) score exceeded six. All the patients routinely received intravenous ondansetron (4 mg) at the end of surgery for prophylaxis of postoperative nausea and vomiting. Postoperative pain was assessed using the VAS and recording postoperative tramadol consumption. The incidence of postoperative nausea and vomiting was monitored by recording the administration of ondansetron in the post-anesthesia care unit or on the day of hospital discharge.

**Figure 3:**
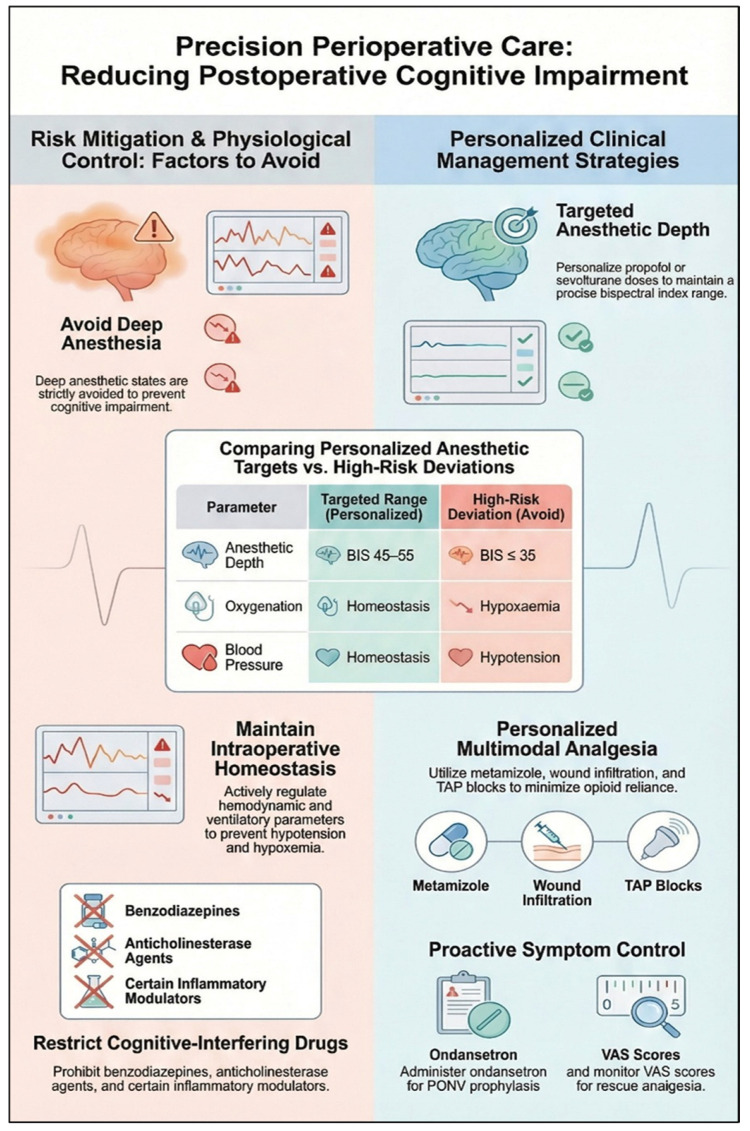
Schematic presentation of anesthetic protocol. Created using NotebookLM^®^ (Google, accessed in 9 March 2026).

Perioperative factors associated with postoperative cognitive impairment were actively and strictly controlled intraoperatively, including hypotension, hypoxemia, excessively deep anesthetic depth (BIS ≤ 35), hypothermia, hypercarbia and significant intraoperative bleeding. Patients who developed any of these protocol deviations were prospectively excluded from the study. The use of medications known to modulate inflammatory responses was prohibited throughout the study period, including dexmedetomidine, clonidine, dexamethasone and non-steroidal anti-inflammatory drugs, or drugs that interfere in cognitive outcomes, such as benzodiazepines and anticholinesterase agents. The rationale to this anesthetic protocol is explained in Figure 3.

### 2.7. Demographic and Clinical Data

Demographic and clinical data were recorded, including age, sex, educational level, body surface area (BSA), ASA physical status, comorbidities, medication use, and history of prior surgery. Intraoperative hemodynamic parameters and total surgical or anesthesia durations were extracted from anesthesia records, and postoperative data were collected from the electronic medical record.

Postoperative delirium was screened using the Confusion Assessment Method for the Intensive Care Unit (CAM-ICU), applied exclusively to the randomized surgical groups on the day of hospital discharge. The CAM-ICU is a validated instrument for detecting delirium, characterized by acute changes in mental status and altered levels of consciousness. A positive CAM-ICU result was considered an exclusion criterion [25].

### 2.8. Measurements to Assesses Postoperative Changes

The primary outcome was to assess differences in cognitive performance between the three groups on the day of hospital discharge and baseline, using CVAT parameters (RT, VRT, CV, CE, and OE) and COWAT and SDMT scores. The secondary outcome was to evaluate differences in cognitive performance among the three groups at D15 and D45 after surgery using the same parameters.

To assess postoperative changes in cognitive performance, for the primary outcome, we first calculated a relative change score (ratio) for each variable using a formula that represents the proportion of baseline performance alterations on the first day after surgery.

Primary outcome: (baseline performance − postoperative day 1)/baseline performance.

For the secondary outcome, we used the same equation. This approach allowed the assessment of cognitive performance changes over time relative to baseline using the same directional framework as the primary outcome.

Secondary outcome: (baseline performance − Postoperative Day 15 or Day 45)/baseline performance.

#### Directional Alignment

The interpretation of ratio scores differed by variable type. For OE, CE, RT, and VRT, a negative ratio indicated a decline in performance (i.e., more errors, slower or more variable reaction times), while a positive ratio indicated improvement. For the COWAT and SDMT, the opposite pattern applied: a negative ratio indicated improvement (learning or practicing better performance on the second test), while a positive ratio indicated decline. This directional distinction was carefully considered when interpreting the results.

### 2.9. Sample Size

The sample size was calculated using the formula Np (Np = [(*Z*α/2 + *Z*β). σ/D]2), where α = Type I error; α = 0.05. β level was set at 0.20 and power (which = 1 − β) was 0.80; σ = common standard deviation (based on previous test–retest reliability of CVAT study with 200 subjects) [18]; D = minimum difference accepted (D). The values of these differences were estimated assuming they would reach levels of clinical significance. Based on this consideration, the minimum sample size for the pairwise comparisons was 25 subjects per group. Given the approximately 10% loss to follow-up at each time point (DD, D15 and D45), we estimated that 34 participants per group would be required for the primary outcome. A formal MANOVA-based sample size calculation was not conducted because reliable a priori estimates of the multivariate covariance structure among the six cognitive outcome variables were not available. MANOVA was employed as an omnibus test to evaluate whether the intervention produced a significant overall effect across the set of cognitive outcomes while controlling the experiment-wise Type I error rate. Only after establishing a significant multivariate effect were univariate ANOVAs examined to identify the specific cognitive domains contributing to the overall multivariate finding. Consequently, the univariate analyses should be viewed as protected follow-up tests rather than independent primary outcomes requiring separate sample size calculations.

### 2.10. Statistical Analysis

#### 2.10.1. Baseline and Clinical Variables

As the non-surgical group was not included in the randomization process, differences in demographic, variables and baseline neurocognitive test raw scores among the three groups were compared using one-way univariate analyses of variance (ANOVAs). Intraoperative and postoperative variables were compared among the groups using independent-samples *t*-tests for continuous variables and chi-square tests for categorical variables, that included duration of surgery, duration of anesthesia, minimum BIS, minimum heart rate, minimum mean arterial pressure, SpO_2_, and postoperative pain intensity (assessed by opioid consumption on the day of hospital discharge), and the occurrence of postoperative nausea and vomiting, as indicated by antiemetic administration, was evaluated. Pairwise comparison using ANOVA was performed between the non-surgical propofol and sevoflurane groups with VAS postoperative pain scores.

#### 2.10.2. Multivariate Analysis

To determine whether declines were specific to the surgical groups, we performed a two-tailed multivariate analysis of variance (MANOVA) comparing the three groups (non-surgical, propofol, sevoflurane) on the ratio scores of all six variables. A significant multivariate effect (*p* < 0.05) would indicate an overall group difference in performance change and justify proceeding to univariate analyses. All assumptions for MANOVA were examined prior to analysis. Homogeneity of covariance matrices was assessed using Box’s M test (*p* > 0.001), and no serious violations were detected.

#### 2.10.3. Univariate Follow-Up

Following the MANOVA, we conducted univariate ANOVAs on each variable. Given our directional hypothesis that the surgical groups would show greater declines than the non-surgical group, we applied one-tailed tests for all variables. For OE, CE, RT, and VRT, the one-tailed hypothesis predicted that the surgical groups would have worse ratio scores than surgical, reflecting greater decline. For the COWAT and SDMT, the one-tailed hypothesis predicted that the surgical groups would have worse scores than the non-surgical groups. To control for Type I error due to multiple comparisons, we applied a Bonferroni correction. The use of one-tailed follow-up tests was prespecified and based on a directional hypothesis derived from the postoperative cognitive dysfunction literature. The primary research question was whether the patients undergoing surgery under general anesthesia would exhibit greater cognitive decline than non-surgical controls. No plausible theoretical or empirical rationale supported the opposite direction of effect, namely cognitive improvement attributable to surgery or anesthesia in the immediate postoperative period. Therefore, after establishing a significant overall multivariate effect through a two-tailed MANOVA, one-tailed univariate analyses were used to test the specific *a priori* hypothesis of postoperative cognitive deterioration.

#### 2.10.4. Pairwise Comparisons

For variables with significant univariate effects, we performed pairwise comparisons with Bonferroni adjustment to identify specific group differences.

Statistical significance was defined as *p* < 0.05, consistent with the directional hypotheses established in the primary outcome analysis. All the analyses realized in the study were performed using SPSS version 31.0.0.0 for Windows (SPSS Inc., Chicago, IL, USA).

## 3. Results

### 3.1. Participants

**Figure 4:**
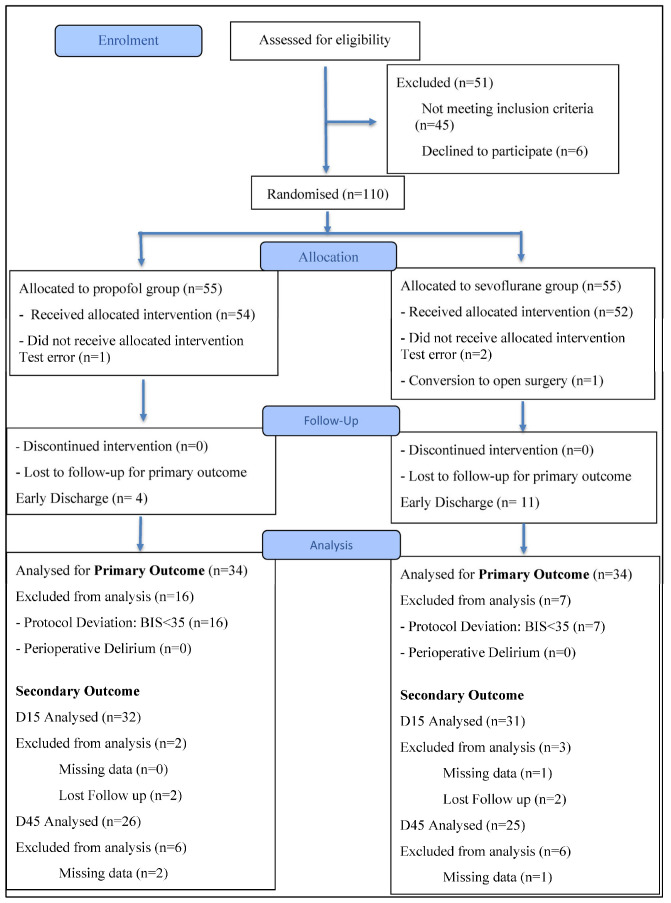
CONSORT 2025 Flowchart Diagram.

From September 2023 to September 2024, 110 patients were enrolled in the intervention groups and allocated to groups randomly (Figure 4). Of these, 68 patients completed the trial and had primary outcome data available at discharge, with 34 in the sevoflurane group and 34 in the propofol group. In the non-surgical group of the study, 55 participants were screened, of whom 37 were eligible and included in the primary outcome analysis. As for the secondary outcomes: 28 participants remained eligible at D15 and 25 at D45 in the non-surgical group due to loss to follow-up.

### 3.2. Demographic Data and Clinical Variables

The sample showed no significant differences in age, ASA, BSA, education and sex distribution were observed among the groups (Table 1). No significant difference in VAS pain or neuropsychological test scores was observed among the three groups at baseline (Table 1).

At hospital discharge, the mean VAS on discharge day in ANOVA showed significant difference among the groups (Table 1); in pairwise comparisons both the surgical groups had significantly higher pain scores than the non-surgical group (propofol vs. non-surgical: *p* < 0.001; sevoflurane vs. non-surgical: *p* < 0.001). No significant difference was observed between the propofol and sevoflurane groups (*p* = 0.505). Additionally, no patient screened positive for delirium on the CAM-ICU in any group.

As shown in Table 2, intraoperative hemodynamic parameters, length of hospital stay, total surgical and anesthesia durations, and postoperative outcomes were similar between the propofol and sevoflurane groups.

### 3.3. Primary Outcomes

#### 3.3.1. Multivariate Analysis of Performance Changes

For the discharge day, the MANOVA revealed a significant multivariate effect of group; Pillai’s Trace = 0.332, F (12, 196) = 3.246, *p* < 0.001, and partial η^2^ = 0.196, indicating that the pattern of performance changes differed significantly across the three groups. This moderate-to-large effect size justified proceeding to univariate analyses.

#### 3.3.2. Univariate Analysis for the Day of Hospital Discharge

Following the significant MANOVA, one-tailed univariate ANOVAs were conducted on each dependent variable. For OE, no significant group differences were observed (*p* = 0.340). For CE, univariate ANOVA approached reach significance (*p* = 0.049) and should be interpreted as a trend. RT showed no significant group differences (*p* = 0.145). VRT showed a significant effect, (*p* = 0.025). This indicates that anesthesia significantly affected the consistency of reaction time performance. COWAT demonstrated a significant effect of group (*p* = 0.002). The SDMT demonstrated a significant effect of group (*p* = 0.005).

#### 3.3.3. Pairwise Comparisons

For VRT, the non-surgical group showed slight improvement, while both surgical groups demonstrated increased variability, indicating poorer sustained attention. Pairwise comparisons with Bonferroni adjustment revealed that the propofol group showed a more significant decline in VRT (increased variability) than the non-surgical group (*p* = 0.034). The sevoflurane group showed a non-significant trend to a worse performance than the non-surgical group (*p* = 0.088). No significant difference was observed between propofol and sevoflurane (*p* = 0.500).

**Figure 5:**
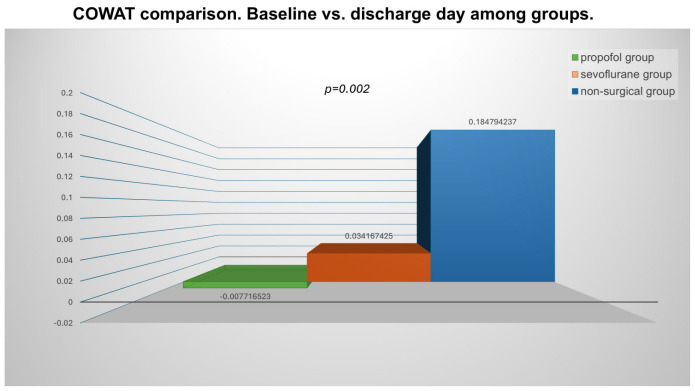
A COWAT comparison. Baseline vs. discharge day among the groups. Note: The data present the delta discharge COWAT values on discharge day using the formula [(−) [(baseline performance—discharge day performance)/baseline performance]], which showed a significant improvement among groups in ANOVA (*p* = 0.002), and a significant improvement in non-surgical groups when compared with the sevoflurane (*p* = 0.021) and propofol (*p* = 0.003) groups in pairwise comparisons.

For the COWAT (Figure 5), the non-surgical group showed a clear improvement, while the propofol and sevoflurane groups demonstrated no significant improvements. Pairwise comparisons with Bonferroni adjustment revealed that the propofol group showed a more significant decline (poor performance) than the non-surgical group (*p* = 0.003). The sevoflurane group also differs significantly from the non-surgical group, showing worse performance (*p* = 0.021). No significant difference was observed between the propofol and sevoflurane groups (*p* = 0.500).

For the SDMT, the non-surgical group showed a clear improvement and the propofol and sevoflurane groups presented no improvement. Pairwise comparisons with Bonferroni adjustment revealed that the non-surgical group showed significant improvement (better performance) than the sevoflurane group (*p* = 0.004). Non-surgical group showed a non-significant trend to an improved performance when compared to the propofol group (*p* = 0.103). No significant difference was observed between the propofol and sevoflurane groups (*p* = 0.347).

### 3.4. Secondary Outcomes

#### Multivariate Analysis of Performance Changes—D15 and D45

For D15, the MANOVA revealed a non-significant multivariate effect of group; Pillai’s Trace = 0.067, F (12, 168) = 0.484, *p* = 0.922, and partial η^2^ = 0.168. For D45, the MANOVA revealed a non-significant multivariate effect of group; Pillai’s Trace = 0.093, F (12, 140) = 0.571, *p* = 0.862, and partial η^2^ = 0.140. In both, the pattern of performance changes did not differ significantly across the three groups. These results did not justify proceeding to univariate analyses.

## 4. Discussion

At discharge day, as the primary outcome, sustained attention was affected in the surgical groups. In terms of a practice effect on the COWAT and SDMT, statistically significant differences in performance were observed between the non-surgical group and both surgical groups. The propofol and sevoflurane groups did not differ significantly on any neurocognitive test. In the secondary outcome, at D15 and D45, no significant difference was observed among the three groups.

An important finding of this study is the significant effect of anesthesia on VRT, a measure of sustained attention and response consistency. The propofol group showed significantly increased VRT, indicating poorer sustained attention, while the sevoflurane group showed a trend in the same direction. The sevoflurane group’s intermediate position (worse than non-surgical but better than the propofol group, though not significantly different from either) suggests that inhalation anesthesia may confer relative protection of attentional function. This pattern is consistent in other studies, where propofol produced actual decline while sevoflurane produced only diminished learning [26,27]. Nevertheless, the direct comparison between propofol and sevoflurane did not reach statistical significance. The importance of these findings highlights the risk for attentional impairment in patients undergoing minor surgical procedures at the time of hospital discharge, as well as the possible need for neuropsychological assessment at this moment.

VRT is considered a pure measure of sustained attention, relatively free from the influence of motor speed or cognitive strategy [18,19,20,21,22,23,24]. Propofol and sevoflurane selectively impaired this function, suggesting that general anesthesia may have specific effects on attentional networks, potentially involving thalamocortical circuits or frontal attention systems (PFC) [28]. This mechanism involves increased release of noradrenaline and dopamine, leading to activation of lower-affinity adrenoceptors and a consequent reduction in neuronal firing within the PFC, which plays a central role in sustaining attention and integrating executive process [29]. These findings are consistent with previous evidence demonstrating transient modulatory effects of both propofol and sevoflurane on prefrontal cortical activity during general anesthesia, typically lasting a few hours and resolving thereafter [29]. Both agents reduce prefrontal metabolism and frontoparietal connectivity, which may result in slower information processing, which may affect increasing VRT [30]. Such transient disruption of prefrontal networks provides a plausible neurobiological explanation for the short-lived impairment in sustained attention observed at hospital discharge in the present study.

Persistent postoperative pain could affect the VRT, considering pain has long been correlated with poor attentional regulation [31,32,33]. In line with this interpretation, both surgical groups exhibited higher pain scores at discharge compared with the non-surgical group, with no difference between the propofol and sevoflurane groups, as expected [31]. The inducement of moderate acute pain may affect attention and is sometimes a discharge risk [32,33].

In contrast to VRT, RT did not differ between the preoperative and discharge measurements in any group. In this context, this dissociation between mean speed (RT) and consistency is clinically meaningful. Patients may maintain their average processing speed while becoming more inconsistent in their responses, reflecting lapses of attention rather than generalized slowing [21,23]. This finding corroborates prior research demonstrating increased VRT following sustained attention tasks, without significant changes in RT in patients with pain (fibromyalgia [31] and spinal [34].

The clinically significant differences in COWAT performance between the non-surgical group and both surgical groups were driven by greater improvement in the non-surgical group, suggesting a practice effect restricted to this group [15]. COWAT tasks require not only lexical access and executive function but also sustained attention to maintain task focus throughout the testing period [15]. The absence of learning or practice effects in the surgical groups may be partially explained by impaired sustained attention, as reflected in increased VRT. Patients with poorer attention are less able to benefit from previous exposure to the task, resulting in diminished or absent practice effects.

Although the study explored whether propofol- and sevoflurane-based anesthesia would be associated with different postoperative cognitive profiles, our findings did not demonstrate significant differences between these two groups. This result should be interpreted in the context of the conflicting evidence available in the literature [35,36,37,38,39].

The same happens with the SDMT, which demonstrated a better performance in the non-surgical groups in comparison to the surgical groups with statistical significance in the sevoflurane group, and a trend in the propofol group [16]. These findings are in line with earlier studies that documented early postoperative cognitive changes after propofol and sevoflurane anesthesia [26,27]. At postoperative days 15 and 45, a learning effect was noted in both the surgical and non-surgical groups, consistent with prior reports [37,38].

Although meta-analyses and systematic reviews have proposed a potential neuroprotective role of propofol in postoperative cognitive outcomes [4,12], the present study found no significant differences between propofol and sevoflurane regarding cognitive endpoints, contrary to the initial hypothesis of a protective effect of propofol. Additionally, recent well-designed studies [13] have also failed to demonstrate statistically significant differences between these two anesthetic approaches. Even though earlier controversial reports described greater impairment in the propofol group [14], our results indicate comparable cognitive performance between propofol and sevoflurane, with no statistically significant differences. However, this study was not formally powered for the three-group MANOVA framework or for detecting subtle differences between propofol and sevoflurane anesthesia.

Our study limitations comprehend the application of simplified neurocognitive assessment battery due to logistic limitations: in the COWAT we only used the phonetic form evaluation, which can be affected by education and cultural background [15]; in the CAVT we used a small version of five minutes, instead of fifteen minutes [18]; in the SDMT, we applied the test in smaller intervals than forty-five days, where a test–retest learning effect has been observed, which might hinder its capability to detect earlier cognitive changes [16,17].

A second limitation is that the sample size calculation was based on the anticipated between-group differences in cognitive performance derived from prior studies. A formal MANOVA-based sample size estimation was not performed because reliable estimates of the covariance structure among the multiple cognitive outcomes were unavailable at the design stage. However, the MANOVA yielded a partial η^2^ of approximately 0.20, corresponding to a Cohen’s f of approximately 0.49, which represents a moderate-to-large effect size. Given the final sample size of 105 participants distributed across three groups, the achieved statistical power for detecting the observed multivariate effect was high, indicating that the study was adequately powered for its primary multivariate analysis.

Another limitation of ours was a relatively small sample size due to losses to follow-up, particularly on postoperative days 15 and 45. Such attrition may have reduced the statistical power to detect subtle or late-stage cognitive changes, emphasizing the need for further studies with larger samples. In addition, post-randomization exclusion due to BIS < 35 may have affected group balance and introduced selection bias, particularly because exclusions were asymmetrically distributed between surgical groups. Finally, the validity of one-tailed follow-up tests should be interpreted cautiously given the multiple cognitive endpoints and the exploratory nature of the analyses.

Our strengths included the use of the CVAT; this method used the objective outcomes measured at the end of each of these days to limit examiner bias and the risk of learning effects, which has been a major strength of this study [40]. The CVAT provides an objective measurement of attentional status, has been shown to be relatively immune to the effect of educational level and is free from cultural bias, providing evidence for its application to different population groups [23,24]. The standardization of the anesthetic technique, meticulous control of perioperative factors, and the exclusion of inflammatory modulators reduce the bias of this study; the inclusion of a non-surgical comparison group was especially useful as it allowed us to identify a learning effect that was not present in the surgical patients.

The present study represents one component of a broader approved research protocol. Future investigations will analyze the remaining variables included in the original protocol, such as biomarker analyses, including S100β assessment, as well as additional neuropsychological evaluations.

## 5. Conclusions

Minor surgery under general anesthesia may lead to transient impairments in attention and learning at the time of hospital discharge. No significant differences were observed between the propofol and sevoflurane groups, although, exploratory analysis suggested postoperative cognitive changes in the surgical patients compared with the non-surgical group. These findings support the potential value of postoperative cognitive monitoring and individualized discharge decisions.

## Figures and Tables

**Table 1:** A comparative analyses of demographic, clinical data and baseline neurophysiological performance among the propofol, sevoflurane and non-surgical groups.

Variable	Non-Surgical Group (*n* = 37)	Propofol Group (*n* = 34)	Sevoflurane Group (*n* = 34)	*p* Value
Demographic Data				
Age (years)	48.54 ± 16.8	53.27 ± 14	55.85 ± 14.02	0.145
Females (n, %)	25 (75.60%)	29 (85.28%)	28 (85.30%)	0.120
BSA (m^2^)	1.88 ± 0.28	1.84 ± 0.21	1.90 ± 0.25	0.515
ASA Physical Status				
1 (n, %)	21 (56.75%)	16 (47.05%)	13 (38.23%)	0.302
2 (n, %)	16 (43.25%)	18 (52.94%)	21 (61.76%)	0.302
Education (years)	10.68 ± 3.12	9.74 ± 3.71	9.56 ± 3.31	0.299
VAS Discharge Day	0.667 ± 1.25	3.00 ± 2.83	3.28 ± 2.97	**0.001**
Baseline Neurophysiological Performance				
RT	459.24 ± 46.03	466.50 ± 62.14	471.32 ± 62.13	0.636
VRT	97.46 ± 25.06	97.82 ± 35.23	101.21 ± 44.77	0.911
OE	1.11 ± 1.58	2.09 ± 2.82	1.68 ± 2.46	0.168
CE	3.00 ± 2.73	2.62 ± 2.22	2.56 ± 2.11	0.727
COWAT	29.70 ± 10.66	27.97 ± 11.70	29.68 ± 12.93	0.781
SDMT	29.70 ± 12.01	28.68 ± 10.85	28.53 ± 14.96	0.910

Note: The data are presented as means ± SD for the continuous variables or numbers (n, %) for categorical variables. *p*-value was calculated with ANOVA. Bold values indicate statistical significance at the *p* < 0.05 level. Abbreviations: BSA: Body Surface Index (m^2^); y: years; RT, reaction time (milliseconds); VRT, variability reaction time (milliseconds); OE, omission error; CE, commission error; COWAT, controlled oral word association test; SMDT, symbol digit modalities test.

**Table 2:** Comparative analyses of the intraoperative and postoperative data between the propofol and sevoflurane groups. Note: The data are presented as means ± SD for the continuous variables or numbers (n, %) for categorical variables. *p*-value was calculated with *t*-test or X^2^. Abbreviations: BIS, Bispectral Index; MAP, mean arterial pressure; HR, heart rate; PONV, postoperative nausea and vomiting.

Variable	Propofol Group (*n* = 34)	Sevoflurane Group (*n* = 34)	*p* Value
Intraoperative Data			
Duration of surgery (min)	100.0 ± 39.7	89.1 ± 33.5	0.226
Duration of anesthesia (min)	167.2 ± 44.1	158.5 ± 40.5	0.398
BIS values (minimum)	41.0 ± 6.4	42.4 ± 4.7	0.295
MAP (mmHg)	108.7 ± 18.7	110.0 ± 19.0	0.775
HR (min)	61.9 ± 9.5	64.4 ± 10.5	0.336
SpO_2_ (%)	99.9 ± 0.2	100.0 ± 0.0	0.169
Cutaneous temperature; °C	36.0 ± 0.26	36.0 ± 0.27	0.616
EtCO_2_; mmHg	32.8 ± 5.79	32.5 ± 3.78	0.843
Length of hospital stay	1.32 ± 0.475	1.38 ± 0.493	0.618
Postoperative Data			
Postoperative opioids consumption	2 (5.8%)	3 (8.8%)	0.642
PONV	5 (14.70%)	3 (8.8%)	0.452

## Data Availability

The original contributions presented in this study are included in the article. Further inquiries can be directed to the corresponding author.

## Abbreviations

The following abbreviations are used in this manuscript:

CVAT	Computerized Visual Attention Test
COWAT	Controlled Oral Word Association Test
SDMT	Symbol Digit Modalities Test
SDD	Same-Day Discharge
ReBEC	Brazilian Clinical Trials Registry
RBR-6xhp399	Trial registration ID (ReBEC)
UTN	code (not expanded in the text)
U1111-1295-3640	UTN trial code
CAAE	Ethics committee ID code (not expanded in the text)
No.	Number (as in “Approval No.”)
CONSORT	Consolidated Standards of Reporting Trials
ASA	American Society of Anesthesia Physical Status
BIS	Bispectral Index
D15	Day 15 (postoperative)
D45	Day 45 (postoperative)
FAS	Phonemic task using letters F, A, S
OE	Omission Errors
CE	Commission Errors
RT	Reaction Time
VRT	(Intra-individual) Variability in Reaction Time
TAP	Transversus Abdominis Plane (block)
VAS	Visual Analog Scale
BSA	Body Surface Area
CAM-ICU	Confusion Assessment Method for the Intensive Care Unit
Np	Used in sample size formula (not defined in the text)
Zα/2	Z value for alpha/2
Zβ	Z value for beta
α	*Type I error (alpha)*
β	Beta level
σ	Common standard deviation
D	Minimum accepted difference
SpO_2_	Peripheral Oxygen Saturation
MANOVA	Multivariate Analysis of Variance
ANOVA/ANOVAs	Analysis of Variance
SPSS	Statistical Package for the Social Sciences
Inc.	Incorporated
IL	Illinois
USA	United States of America
df	Degrees of Freedom
*p*	*p*-value
F	F statistic
η^2^/η^2^	Eta Squared (effect size)
M	Mean
SE	Standard Error
ms	milliseconds

## References

[1] Cibelli M., Brodier A.E. (2021). Postoperative cognitive dysfunction in clinical practice. BJA Educ..

[2] Luo R., Qiu M., Wu W. (2025). Effects of volatile and intravenous anesthetics on postoperative cognitive dysfunction: A mechanistic review. J. Anesth..

[3] Gan T.J., Ramirez M.F. (2023). Total intravenous anesthesia versus inhalation anesthesia: How do outcomes compare?. Curr. Opin. Anesthesiol..

[4] Miller D., Lewis S.R.E., Printchard M.W., Schofield-Robinson O.J., Shelton C.L., Alderson P., Smith A.F. (2018). Intravenous versus inhalational maintenance of anesthesia for postoperative cognitive outcomes in elderly people undergoing non-cardiac surgery. Cochrane Database Syst. Rev..

[5] Glasbey J.C., Abbott T.E.F., Ademuyiwa A., Adisa A., AIAmeer E., Alshryda S., Arnaud A.P., Kendall B.B., Chaar M.K.A., Chaudhry D. (2022). Elective surgery system strengthening development, measurement, and validation of the surgical preparedness index across 1632 hospitals in 119 countries. Lancet.

[6] Rohi A., Olofsson M.E.T., Jakobsson J.G. (2022). Ambulatory anesthesia and discharge: An update around guidelines and trends. Curr. Opin. Anesthesiol..

[7] Safavynia S.A., Goldstein P.A. (2019). The Role of Neuroinflammation in Postoperative Cognition Dysfunction: Moving from Hypothesis to Treatment. Front. Psychiatry.

[8] Lui Y., Yang W., Xue J., Chen J., Lui S., Zhang S., Zhang X., Gu X., Dong Y., Qiu P. (2023). Neuroinflammation: The central enabler of postoperative cognitive dysfunction. Biomed. Pharmacother..

[9] Inada T., Hirota K., Shingu K. (2015). Intravenous anesthetic propofol suppresses prostaglandin E_2_ and cysteinyl leukotriene production and reduces edema formation in arachidonic acid-induced ear inflammation. J. Immunotoxicol..

[10] Irwin M.G., Chung C.K.E., Ip K.Y., Wiles M.D. (2020). Influence of propofol-based total intravenous anesthesia on peri-operative outcome measures: A narrative review. Anaesthesia.

[11] Wang C.M., Chen W.C., Zhang Y., Lin S. (2021). Update on the Mechanism and Treatment of Sevoflurane-Induced Postoperative Cognitive Dysfunction. Front. Aging Neurosci..

[12] Zhou J., Zhang C., Fang X., Zhang N., Zhang X., Zhu Z. (2023). Activation of autophagy inhibits the activation of NLRP3 inflammasome and alleviates sevoflurane-induced cognitive dysfunction in elderly rats. BMC Neurosci..

[13] Guo L., Lin F., Dai H., Du X., Yu M., Zhang J., Huang H., Ge W., Tao G., Pan L. (2020). Impact of Sevoflurane Versus Propofol Anesthesia on Post-Operative Cognitive Dysfunction in Elderly Cancer Patients: A Double-Blinded Randomized Controlled Trial. Med. Sci. Monit..

[14] Negrini D., Wu A., Oba A., Harnke B., Ciancio N., Krause M., Clavijo C., Al-Musawi M., Linhares T., Bustamante A.F. (2020). Incidence of Postoperative Cognitive Dysfunction Following Inhalational vs Total Intravenous General Anesthesia: A Systematic Review and Meta-Analysis. Neuropsychiatr. Dis. Treat..

[15] Ross T.P., Calhoun E., Cox T., Wenner C., Kono W., Pleasant M. (2007). The reliability and validity of qualitative scores for the Controlled Oral Word Association Test. Arch. Clin. Neuropsychol..

[16] Strober L.D.J., Benedict R.H.B., Jacobs A., Cohen J.A., Chiravalloti N., Hudson L.D., Rudick R.A., LaRocca N.G. (2019). Symbol Digit Modalities Test: A valid clinical trial endpoint for measuring cognition in multiple sclerosis. Mult. Scler. J..

[17] Pereira D.R., Costa P., Cerqueira J.J. (2015). Repeated Assessment and Practice Effects of the Written Symbol Digit Modalities Test Using a Short Inter-Test Interval. Arch. Clin. Neuropsychol..

[18] Schmidt J., Senges G.S., Campos R.G.F., Costa G.L.A., Boechat Y.E.M., Leite J.C.B., Portela A.S., Lewandrowski K.U., Lacerda G.C.B., Schmidt G. (2024). Sustained attention can be measured using a brief computerized attention task. Sci. Rep..

[19] Simões E.M., Carvalho A.L.N., Schmidt S.L. (2017). What does handedness reveal about ADHD? An analysis based on CPT performance. Res. Dev. Disabil..

[20] Filho A.C., Duinkerken E.V., Tolentino J.C., Schmidt S.L. (2022). Attention profile of physically recovered COVID-19 inpatients on the day of discharge. J. Psychiatr. Res..

[21] Simões E.M., Padilha C.S., Bezerra M.S., Schmidt S.L. (2018). Analysis of attention Subdomains in obstructive Sleep Apnea Patients. Front. Psychiatry.

[22] Abramovicz C.C., Fernandes M.M., Senges G.S., Schmidt S.L. (2025). End-stage kidney disease patients exhibited slower responses to rapidly presented visual stimuli when compared with healthy controls. Neuropsychology.

[23] Schmidt S.L., Schmidt G.J., Padilha C.S., Simões E.M., Tolentino J.C., Barros P.R., Narciso J.H., Godoy E.S., Filho R.L.C. (2019). Decrease in Attentional Performance After Repeated Bouts of High-Intensity Exercise in Association-Football Referees and Assistant Referees. Front. Psychol..

[24] Duinkerken E.V., Schmidt G.J., Gjourp A.L.T., Mello C.R., Marques A.C., Filho A.C., Fukusawa P.R.Y., Assis S.G., Tolentino J.C., Schmidt S.L. (2021). Assessment of Attentional Functioning in Health Professionals of a Brazilian Tertiary Referral Hospital for COVID-19. Behav. Neurol..

[25] Miranda F., Gonzalez F., Plana M.N., Zamora J., Quinn T.J., Seron P. (2023). Confusion Assessment Method for the Intensive Care Unit (CAM-ICU) for the diagnosis of delirium in adults in critical care settings. Cochrane Database Syst. Rev..

[26] Zech N., Seemann M., Luerding R., Doenitz C., Zeman F., Cananoglu H., Kees M.G., Hansen E. (2021). Neurocognitive Impairment After Propofol with Relevance for Neurosurgical Patients and Awake Craniotomies—A Prospective Observational Study. Front. Pharmacol..

[27] Kletecka J., Holeckova I., Brenkus P., Pouska J., Benes J., Chytra I. (2019). Propofol versus sevoflurane anesthesia: Effect on cognitive decline and event-related potentials. J. Clin. Monit. Comput..

[28] Sanger J., Bechtols L., Schoofs D., Blaszkewicz M., Wascher E. (2014). The influence of acute stress on attention mechanisms and its electrophysiological correlates. Front. Behav. Neurosci..

[29] Flores F.J., Hartnack K.E., Fath A.B., Kim S.E., Wilson M.A., Brown E.N., Purdon P.L. (2017). Thalamocortical synchronization during induction and emergence from propofol-induced unconsciousness. Proc. Natl. Acad. Sci. USA.

[30] Chang E., Wang Y., Zhu R., Wu L., Yang Y., Zeng S., Li N., Ruan X., Sun M., Zhang W. (2023). General anesthetic action profile on the human prefrontal cortex cells through comprehensive single-cell RNA-seq analysis. iScience.

[31] Schmidt G., Alvarenga R., Manhães A., Schmidt S. (2017). Attentional Performance may help to identify duloxetine responders in chronic pain fibromyalgia patients. Eur. J. Pain.

[32] Pokkinen S.M., Yli-Hankala A., Kalliomäki M.L. (2014). The effects of propofol vs. sevoflurane on post-operative pain and need of opioid. Acta Anaesthesiol. Scand..

[33] Vancleef L.M.G., Peters M.L. (2006). Interruptive Effect of pain on attention. J. Pain.

[34] Lewandrowski K.U., Blum K., Lorio M., Hana C., Lewandrowski A.P.L., Lundquist T., Fiorelli R.K.A., Schmidt G.J., Liodakis E., Gold M. (2026). Sustained Attention Instability as a Cognitive Biomaker in Chronic Spine Pain: A 90-Second Visual Attention Test. Eur. J. Pain.

[35] Amare M., Mc Evoy M., Smith A. (2019). The effect of intravenous and inhalational maintenance of anaesthesia on postoperative cognitive outcomes in elderly people. Anaesthesia.

[36] Au E., Thangathurai G., Saripell A., Yan E., Englesakis M., Nagappa M., Chung F. (2023). Postoperative outcomes in elderly patients undergoing cardiac surgery with preoperative cognitive impairment: A systematic review and meta-analysis. Anesth. Analg..

[37] Mohanty S., Gillio A., Lindroth H., Ortiz D., Holler E., Azar J., Boustani M., Zarzaur B. (2022). Major surgery and long-term cognitive outcomes: The effect of postoperative delirium on dementia in the year following discharge. J. Surg. Res..

[38] Cao S.J., Zhang Y., Zhang Y.X., Zhao W., Pan L.H., Sun X.D., Jia Z., Ouyang W., Ye Q.S., Zhang F.X. (2023). Delirium in older patients given propofol or sevoflurane anaesthesia for major cancer surgery: A multicentre randomised trial. Br. J. Anaesth..

[39] Konishi Y., Evered L.A., Scott D.A., Silbert B.S. (2018). Postoperative cognitive dysfunction after sevoflurane or propofol general anesthesia in combination with spinal anesthesia for hip arthroplasty. Anaesth. Intensive Care.

[40] Borchers F., Spies C.D., Freinkohl I., Brockhaus W.R., Kraft A., Kozma P., Fislage M., Kuhn S., Ionescu C., Speidel S. (2021). Methodology of measuring postoperative cognitive dysfunction: A systematic review. Br. J. Anaesth..

